# Eye Health and Vision Function in Adults Aging with Well-Controlled HIV

**DOI:** 10.3390/v18040431

**Published:** 2026-04-02

**Authors:** Alison G. Abraham, Xinxing Guo, Srijana Lawa, Aleks Mihailovic, Michael W. Plankey, Todd T. Brown, Joseph B. Margolick, Pradeep Ramulu, Seema Banerjee

**Affiliations:** 1 Department of Epidemiology, Colorado School of Public Health, University of Colorado, Anschutz Medical Campus, Aurora, CO 80045, USA; 2 Department of Ophthalmology, University of Colorado, Anschutz Medical Campus, Aurora, CO 80045, USA; 3 Department of Epidemiology, Johns Hopkins Bloomberg School of Public Health, Baltimore, MD 21205, USAtbrown27@jhmi.edu (T.T.B.); 4 Department of Ophthalmology, Johns Hopkins School of Medicine, Baltimore, MD 21287, USA; xguo11@jhu.edu (X.G.); amihail2@jhu.edu (A.M.); pramulu@jhmi.edu (P.R.);; 5 Department of Medicine, Georgetown University Medical Center, Washington, DC 20057, USA; mwp23@georgetown.edu; 6 Department of Molecular Microbiology and Immunology, Johns Hopkins Bloomberg School of Public Health, Baltimore, MD 21205, USA; jmargol1@jhu.edu

**Keywords:** HIV, vision loss, eye disease, aging health

## Abstract

Here, we describe vision health in aging adults living with HIV (PLWH) and comparable people without HIV (PWOH) from the MACS/WIHS Combined Cohort Study (MWCCS). PLWH and PWOH aged 60 years and older were recruited from Baltimore/Washington, DC, from September 2021 to September 2023. Exact matching and sample weights were used to create age-balanced comparisons. Visual impairment (VA worse than 20/40 after refraction or CS worse than 1.50 logCS in the better eye) and the presence of eye pathology were assessed. We studied 74 PLWH (97% virally suppressed) and 65 PWOH, aged 61 to 79 years, 36% Black, and 87% male. For PLWH and PWOH, distance VA impairment was noted in 4% vs. 1%, respectively, and uncorrected refractive error in 15% vs. 5%. More than half had signs of dry eye disease (63% for PLWH and 51% for PWOH). About half of PLWH had developed at least an early stage of cataract, compared to 20% of PWOH. Posterior chamber abnormalities were observed in 4% and 0%, and glaucomatous changes in 19% and 25% of PLWH and PWOH, respectively. The need for eyecare was high among this sample of PLWH with viral suppression and PWOH.

## 1. Introduction

Vision loss is highly prevalent in older adults [1,2,3,4] and results in functional declines that directly impact quality of life [5,6,7,8]. People living and aging with HIV are enjoying longer lives, thanks to effective antiviral therapies, but appear to experience more frequent and earlier age-related comorbidities, possibly as a result of long-term exposure to inflammation [9,10,11]. Studies have demonstrated a higher risk of many age-related comorbidities among adults aging with HIV compared to similarly aged and comparable individuals without HIV [12,13]; the same excess risk may extend to age-related eye disease and accompanying vision loss.

Unfortunately, we know very little about the burden of eye disease and vision loss in the modern antiretroviral therapy era with its context of well-controlled HIV, as has been highlighted in the literature [14]. Earlier studies established the risk of vision abnormalities and ocular complications in people with AIDS. From data two decades old, adults living with HIV who are severely immunocompromised are at risk for neuroretinal involvement, intraocular inflammation, structural ocular complications, and visual impairment, as well as earlier cataract development, visual field abnormalities, and thinner retinas [15,16,17,18,19]. However, in the era of highly effective antiretroviral therapy, AIDS has become less relevant as rates of antiretroviral use and durable viral suppression have risen [13]. While many age-related chronic diseases are a focus of current research and funding in existing HIV cohorts, age-related eye diseases and vision outcomes have been largely ignored. One recent study suggested that adults aging with HIV are more likely to report vision difficulties than similarly aged adults living without HIV, though objective assessment of vision function was lacking [20]. The population of adults living with HIV in the US is entering ages where the risk of age-related eye disease is significant, and the additional burden of vision loss could be particularly impactful. Vision loss may amplify the effect of other co-existing conditions on daily life [21], and it may contribute to poor engagement in care [22] and poor patient activation [23,24], which could particularly affect antiretroviral medication adherence [22].

Here we report results from the HIV Vision study, an ancillary descriptive study of the Baltimore/Washington, DC, sites of the MACS/WIHS Combined Cohort Study (MWCCS) that conducted eye and vision exams in a sample of adults aging with HIV and a comparison sample of similar aging adults living without HIV. The purpose of this study was to describe the current burden of eye pathology and vision loss in adults aging with HIV in the modern therapy era. These data can inform strategies for screening and integrated vision care, as some of the most common causes of vision loss in aging adults are correctable [3].

## 2. Materials and Methods

### 2.1. Study Setting and Population

Participants 60 years and older from the Baltimore/Washington DC site of the MWCCS, including people living with HIV (PLWH) and people without HIV (PWOH), were invited to a single vision study visit at the Johns Hopkins Wilmer Eye Institute. The study visit occurred from September 2021 to September 2023. The study protocol adhered to the tenets of Helsinki and was approved by the Institutional Review Boards at the University of Colorado, Johns Hopkins University, and Georgetown University. Written informed consent was obtained from all study participants.

### 2.2. Visual Function, Visual Processing Speed and Refractive Error

Participants completed a series of visual function evaluations, including distance presenting visual acuity (VA), distance corrected VA, near presenting VA, and contrast sensitivity (CS). Distance presenting VA was measured in the right and left eyes separately, with the participant’s habitual correction worn, while reading letters from a retro-illuminated Early Treatment Diabetic Retinopathy Study chart (Precision Vision, La Salle, IL, USA). Each participant was asked to start reading at the top line (corresponding with a VA of 20/200), and the total number of letters correctly read was recorded. Near presenting VA was assessed binocularly with usual correction using sentences from the MNRead charts (Precision Vision) following standard procedures [25]. Participants were instructed to read the test sentences aloud as quickly and accurately as possible, with the MNRead charts placed at a viewing distance of 40 cm. Time and errors in completing each sentence were documented. Refraction and distance-corrected VA were measured using an autorefractor with a built-in VA chart (Nidek ARK 560A, Marco Technologies, Somerset, NJ, USA). CS was measured using the MARS letter CS test (The Mars Perceptrix Corporation, Chappaqua, NY, USA). The right and left eyes were tested separately at 40 cm with habitual corrective lenses worn. The number of letters correctly identified was documented. Room lighting was standardized for each test. Data from the better-functioning eye for each aspect of visual function were used in the analysis because the better eye function has been determined to be most influential on physical function and QoL [26].

Visual processing speed when searching for a target under divided visual attention and in the presence or absence of visual clutter was measured using the online Useful Field of View (UFOV) test (Visual Awareness Research Group, Punta Gorda, FL, USA) [27]. Studies indicate that older adults who perform poorly in a useful field of view task are more likely to experience difficulties in visual tasks of everyday living and are at an elevated risk for motor vehicle collision involvement [28]. Participants completed a sequence of evaluations of processing speed, divided attention, and selective attention.

VA impairment (VI) was defined as acuity worse than 20/40 in the better eye for presenting and corrected distance vision and worse than 20/40 binocularly for presenting near vision. CS impairment (CSI) was defined as worse than 1.50 logCS in the better eye. Uncorrected or undercorrected refractive error (URE) was defined as having worse than 20/40 presenting distance VA and better than 20/40 corrected distance VA in either eye.

### 2.3. Eye Examination and OCT Retinal Imaging

Intraocular pressure (IOP) was measured twice in each eye using iCare tonometry (iCare, Raleigh, NC, USA), and the average was taken. Schirmer’s II test was administered to determine the severity of tear insufficiency, with severity defined based on the length of moisture area from the Schirmer II test [29] and categorized as: none (>15 mm), mild (>10 mm, ≤15 mm), moderate (>5 mm, ≤10 mm), and severe (≤5 mm). Signs of dry eye disease included either the presence of mild or more severe tear insufficiency or Meibomian gland dysfunction, based on one or more findings, including blocked orifices, frothy or foamy secretion, secretion of meibum, telangiectasias, or hyperemia. Participants were also asked about eye discomfort.

Participants were eligible for dilation if no contraindications were identified. In most participants, the right eye was dilated using 2.5% phenylephrine (Alcon Vision LLC, Fort Worth, TX, USA) or 1% tropicamide (Somerset Pharma, Mountainside Medical Equipment, Marcy, NY, USA). The left eye was dilated when the right eye was deemed not eligible or when the left eye had distance-corrected VA worse than 20/40 or logCS worse than 1.50. An ophthalmologist performed slit lamp examinations on dilated eyes, focusing on cataract grading using the Wilmer lens opacity grading system [30], identifying other anterior segment diseases, including Meibomian gland dysfunction, corneal abnormalities, active uveitis, and assessing the posterior segment, including vitreous, fundus, and optic nerve. Any ocular pathology was documented. Cataract was defined as either past evidence of cataract surgery, a nuclear cataract grading ≥ 2, a posterior Subcapsular cataract grading ≥ 3 mm or a cortical cataract grading ≥ 4/16.

Macular angiography centered at the fovea and optic nerve head structural scans were captured using RTVue-XR Avanti OCT (Optovue, Fremont, CA, USA) imaging system. Images were exported with machine-processed measurements, including nerve fiber layer (NFL) thickness, superficial plexus vessel density (VD), and the size of the fovea avascular zone (FAZ).

### 2.4. Other Covariates

Demographics data on age, sex at birth (female, male), race, and ethnicity (Black, Hispanic, White, Other) were extracted from the MWCCS cohort database. The educational background was obtained from the MWCCS baseline visit; annual household income, smoking and alcohol use, diabetes mellitus (DM), hypertension status, and health insurance coverage were obtained from the nearest MWCCS visit. PLWH CD4 cell count, HIV viral load, and years of HIV infection were extracted from the nearest visit. Most values were captured within 2 years of the vision exam date, with the following exceptions: one participant’s viral load, one participant’s smoking status, one participant’s income, one participant’s current alcohol use, and one participant’s hypertension were drawn from 2018 and 2019. DM status was considered positive if the participant had two of the following: (1) self-reported ever use of antidiabetic medication, (2) fasting glucose level ≥ 126 mg/dL, (3) hemoglobin A_1c_ concentration ≥ 6.5%, or (4) self-reported diabetes. Hypertension status was considered positive if the participant had any of the following: (1) systolic blood pressure ≥ 130 mmHg; (2) diastolic blood pressure ≥ 80 mmHg; (3) self-reported use of hypertensive medications and self-reported diagnosis. Years of HIV infection under observation were defined as the number of years between the date of entry into the cohort (if seroconversion occurred prior to baseline) or the estimated seroconversion date (if seroconversion occurred after baseline) and the eye exam date. The seroconversion date was estimated as the midpoint between the last known date when the participant was seronegative and the first known date when the participant was seropositive if the gap between those dates was less than 6 years; otherwise, the first known seropositive date was used.

### 2.5. Statistical Analysis

As age is a critical factor driving the risk of eye disease, matching was used to reduce the mean age difference between PLWH and PWOH at the time of the vision assessment in this descriptive study. The R package matchit [31] (R Statistical Software v4.2.2; R Core Team 2022) was used to implement exact matching, and sample weights were used to create age-balanced comparison groups for analysis. All analyses were performed in the weighted dataset.

Descriptive statistics, including weighted means, medians, and percentages, were calculated for vision loss and eye disease prevalence. Missing covariate data at a visit were filled in using data within 5 years of the visit. Participants with missing data on vision and eye health parameters were excluded from individual summary statistics (listwise deletion), though counts of missingness are included in the table footnotes. For the description of OCTA data, we used the Eye Determinants of Cognition study (EyeDOC) participants as the population benchmark, as the EyeDOC sample was drawn from two distinct general aging adult communities and used similar methods to capture vision function and retinal images [32,33,34]. Descriptions of median NFL thickness, VD, and FAZ area were made stratified by age (60 to 69 years, 70 to 79 years, and 80 to 89 years) and HIV status. Statistical comparisons were made in some cases using weighted Chi-squared tests, weighted T tests, median tests, and exact tests, as appropriate.

## 3. Results

Of the 154 participants enrolled in the study, 139 were included in the analysis following age matching across HIV serostatus groups (74 [53.2%] PLWH and 65 [46.8%] PWOH) (Table 1). The mean weighted age of the PLWH and PWOH groups after age matching and weighting was 67.4 years, 57% were non-Hispanic White, and 36% were non-Hispanic Black. Most participants (87%) were male at birth, limiting our ability to stratify by sex. About one-third of the participants had a household annual income of $30,000 or less, and 57% had at least some college education. 13% were current smokers; 72% were current alcohol users. Among the PLWH group, the weighted average duration of HIV infection under observation in the MWCCS was 28 years (IQR: 20 to 38 years), and 97% had a suppressed viral load defined as less than 200 copies/mL, reflecting the well-treated and durably suppressed context of the current MWCCS cohort.

### 3.1. Visual Function

**Figure 1:**
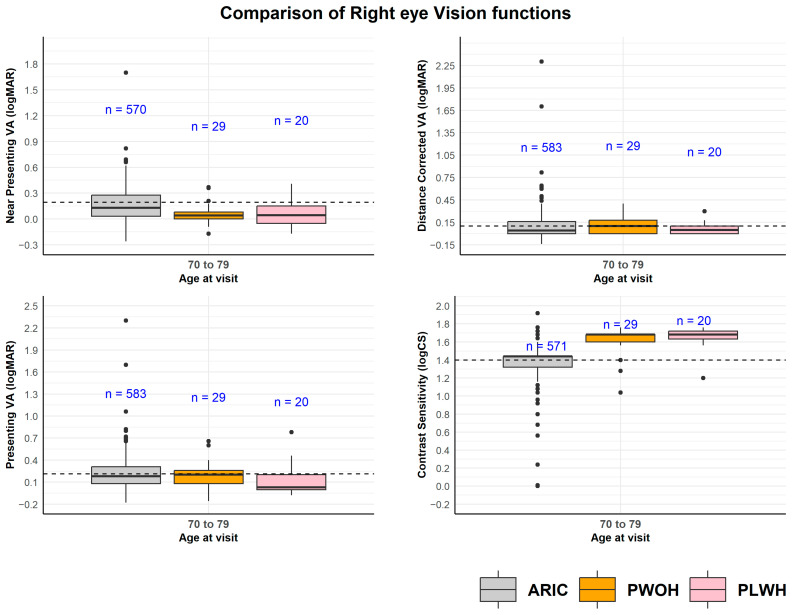
Vision function parameters from vision testing of the right eye. Comparisons of the PLWH and PWOH samples to an aging adult community sample from the EyeDOC study for the age group of 70–79 years. A general aging adult population sample is included from the Atherosclerosis in Communities Study to give a general population age-matched benchmark. The dotted line represents the overall mean value.

The distribution of various indicators of visual function is shown in Figure 1, with an aging adult community sample from the EyeDOC study, which had a mean age of 80 years, shown as a population benchmark [33]. Results are shown for the 70–79 years age strata, as all three samples are represented in this age strata. From Table 2, the prevalence of distance presenting VI in the weighted sample was 4% and 1%, respectively, for PLWH and PWOH. Near-presenting VI was also rare but present more in PLWH than PWOH (9% vs. 4%). For CS, 3% and 4% of PLWH and PWOH had CSI, respectively. URE was more prevalent in PLWH, with 15% (vs. 5% for PWOH) having less than optimally corrected distance acuity. Visual processing speed was equivalent between the PLWH and PLOH groups at 18.4 ms. However, PWOH had slightly higher scores on divided attention (138.7 ms for PLWH vs. 147.4 ms for PWOH), indicating greater difficulty performing multiple tasks simultaneously, while PLWH had higher scores for selective attention (222.1 ms for PLWH vs. 211.4 ms for PWOH), suggesting more difficulty ignoring distractors and focusing on a central target. Comparisons of vision function metrics were tested across HIV groups, but no difference reached statistical significance.

### 3.2. OCTA Findings

**Figure 2:**
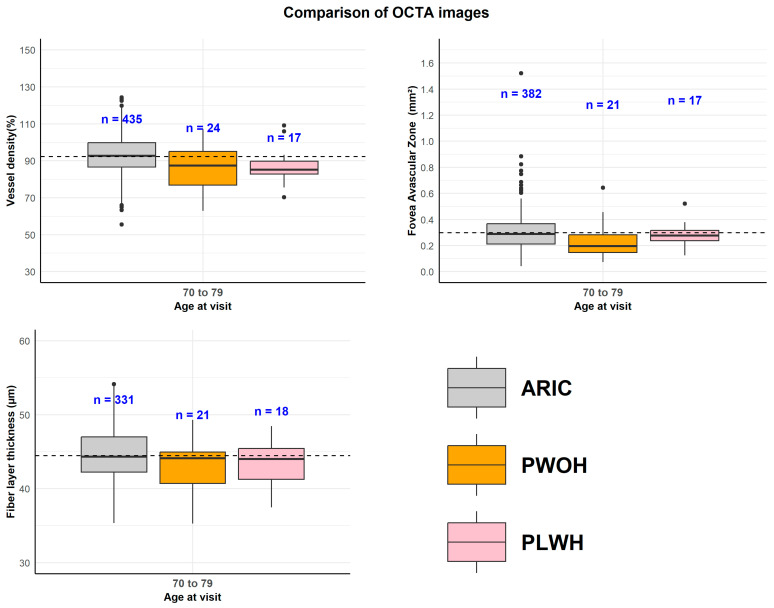
Retinal health parameters from Optical Coherence Tomography images. Comparisons of the PLWH and PWOH samples to an aging adult community sample from the EyeDOC study for the age group of 70–79 years. A general aging adult population sample is included from the Atherosclerosis in Communities Study to give a general population, age-matched comparison. The dotted line represents the overall mean value.

The weighted average optic nerve head NFL thickness was 90 ± 10.7 µm (mean ± SD) for PLWH and 87.5 ± 14.6 µm for PWOH. The weighted superficial capillary plexus VD for the 6 × 6 mm^2^ macular region was 45.1 ± 4.5% and 44.4 ± 3.9% for PLWH and PWOH, respectively. As little data have been published in general population samples to use as a benchmark for OCT-based measures of retinal health, we compared the present data to data from an aging adult community sample in the EyeDOC study, which had a mean age of 80 years [33]. Results are shown in Figure 2 for the 70–79 years age strata, as all three samples are represented in this age strata. The median FAZ among PLWH was larger than that in PWOH but was similar to that in the community sample. Both PLWH and PWOH had lower median RNFL thickness compared to the community sample; the median VD was lower in PLWH compared to the community sample. Comparisons of OCT parameters were tested across HIV groups and between HIV groups and the ARIC sample. Only the lower median VD in PLWH was statistically different from that in the ARIC community sample (*p* = 0.03).

### 3.3. Clinical Findings and Vision Care Access

In PLWH, anterior segment manifestation, including abnormalities of the cornea, iris, ciliary body, and lens, was a common ocular finding. Observed ocular changes included signs of dry eye, conjunctival congestion, corneal endothelial precipitates, corneal scars, superficial punctate keratitis, and iris atrophic changes. Cataract was present in 49% of PLWH and 20% of PWOH, with cortical cataract seen far more often in PLWH (12% versus 1%). Compared to 7% of PWOH, 26% of PLWH had undergone cataract surgery. Meibomian gland dysfunction and other anterior segment abnormalities were apparent in 20% of PLWH participants as opposed to 8% of PWOH participants. Conjunctival congestion and redness were present in 11% of PLWH compared to 4% of PWOH. Thirty-four per cent of the PLWH participants had moderate to severe tear insufficiency, a similar burden to the 33% noted in PWOH. Signs of dry eye disease, defined by meibomian gland dysfunction or mild or worse tear insufficiency, were present in 63% and 51% of PLWH and PWOH, respectively.

Abnormalities in the posterior chamber included retinitis, observed in 4% of the PLWH participants (compared to 0% in PWOH) and glaucomatous changes, including optic disc pallor, optic disc cupping, thinning or notching of neuroretinal rim, observed in 19% of the PLWH participants (compared to 25% of PWOH). Nine per cent of PLWH and 15% of PWOH had an epiretinal membrane. Macular degeneration and diabetic retinopathy were rare in both groups.

For vision care access, 81% and 87% of PLWH and PWOH, respectively, reported having an eye care provider. Access to vision care is highly dependent upon insurance; 57% and 50% of PLWH and PWOH, respectively, reported having private health insurance, and the majority of the remaining participants had Medicare. Despite self-report of generally good access to vision care, 8 participants (6% of the sample; 4 PLWH, 4 PWOH) had high IOP at the time of the HIV Vision Study assessment: 4 reported a glaucoma diagnosis, and 2 reported not having an eye doctor.

## 4. Discussion

Vision health is an important aspect of aging health, given its close links to many domains of functionality that contribute to quality of life [6]. PLWH in the present study who underwent a vision and eye health assessment generally had a low prevalence of VI that was similar to, although slightly higher than, that in PWOH. For example, we found URE to be more prevalent in PLWH (15% vs. 5% for PWOH) after controlling for age differences through matching. Data from the CDC suggest that the prevalence of URE in adults aged 65–79 years is 5%, echoing what we saw among the sample of PWOH [35]. Visual processing speed was equivalent between PLWH and similarly aged PWOH, but PLWH had higher scores for selective attention, which indicates more difficulty ignoring distractors and focusing on a central target.

Numerous studies have found ocular manifestations to be more common in PLWH with low CD4 cell counts [36,37], though the impact on eye disease risk of long-term HIV infection with controlled viral load in the era of highly effective therapy is not well investigated. Underlying mechanisms that could lead to higher prevalence of age-related eye disease and vision dysfunction at earlier ages among PLWH include long-term immune activation [10,11] and mitochondrial dysfunction [38,39]. Ocular tissues are particularly susceptible to inflammation, resulting in vascular and neuronal damage [8,12,13], which can be seen on an ophthalmologic exam. A differential burden of eye disease and vision loss [14,15] in adults aging with HIV compared to age-matched PWOH may implicate chronic immune activation in increasing risk for age-related eye disease and visual dysfunction.

While much attention has been placed on posterior eye dysfunction in PLWH, relatively fewer studies have specifically characterized anterior segment and external ocular disorders. Prior research has found that these disorders affect around one-third of PLWH in the era of highly effective therapy [40,41], with dry eye, the most common anterior segment disorder, found in at least 10% of PLWH [41]. In the present study, the prevalence of signs of dry eye disease was the most common ocular manifestation, affecting 63% and 51% PLWH and PWOH, respectively. In comparison, the US prevalence of symptomatic dry eye was reported by the TFOS DEWS-II report to range from 6.8% in the Physicians Health Study to 21.6% in the Beaver Dam Eye Study [42], suggesting that our sample had an unusually high prevalence of dry eye disease signs. Conjunctival congestion and redness were also noted to be more common among PLWH in our study.

Stable treatment with ART may not reduce the risk of dry eye and other anterior segment disorders among PLWH, as detectable HIV RNA levels have been observed in ocular tissues [43,44,45], even in the absence of a detectable plasma viral load [46], and it has been suggested that HIV impairs and penetrates the blood–retinal barrier by inducing an inflammatory state in retinal pigment epithelium cells through exposure to HIV proteins [47,48]. An autoimmune-like etiology of abnormal tear production, linked to lymphocyte infiltration and ultimately the death of lacrimal gland acini and ducts, may result in a higher prevalence of dry eye in PLWH [49]. As summarized by Nguyen et al. in their paper about Meibomian gland dropout in PLWH, “a comprehensive and quantitative characterization of anterior ocular health in individuals on stable ART is currently lacking.” [50].

Lastly, we noted a higher prevalence of cataract in this sample of well-treated, aging adults living with HIV compared to age-matched PWOH (49% vs. 20%, respectively), with the difference largely driven by a higher prevalence of cortical cataract and a history of cataract surgery. An elevated risk of cataract development has been linked to HIV in the context of AIDS and low CD4 counts [17,51]; the presence of lens opacities in younger adults with HIV has even been suggested as evidence of an accelerated aging phenotype in PLWH [51,52]. Oxidative stress and inflammatory reactions in the eye linked to HIV may accelerate the formation of cataracts [53].

This descriptive study of visual function and eye disease in PLWH and age-matched PWOH has several limitations. Firstly, our study was limited by the size of the sample, as most eye diseases are relatively rare even in aging adults. Hence, the results from this descriptive study are suggestive and should be replicated in larger samples. The sample was also predominantly male, precluding our ability to stratify by sex, as some eye diseases appear to occur more often in women [54]. The MWCCS, while representative of well-treated adults aging with HIV and engaged in care, does not represent the overall population of adults living with HIV in the US. Finally, we were unable to dilate both eyes for all participants for the cataract grading and posterior segment evaluation.

## 5. Conclusions

The results from this descriptive study of vision and eye health in adults aging with and without HIV suggest that there may be a slightly higher burden of vision loss and URE, a higher burden of dry eye and a higher burden of cataract in PLWH compared to age-matched PWOH. Given population estimates from CDC of PLWH over 50 [55] and our estimates of prevalence, this would mean an additional 59,400 PLWH experiencing URE, an additional 71,280 PLWH experiencing dry eye symptoms, and an additional 172,260 PLWH experiencing cataract compared to their PWOH counterparts. Routine ophthalmic examination for aging adults living with HIV could improve detection and diagnosis of correctable vision loss and treatable eye disease [56]. Developing strategies for screening and integrated vision care in conjunction with HIV and aging services could improve the quality of life and functionality among adults aging with HIV.

## Figures and Tables

**Table 1:** Demographics and medical history of the study sample.

	PLWH N = 74	PWOH N = 65
**Demographics and comorbidities**		
Age at HIV Vision Study visit (years), weighted mean (SD)	67.4 (4.7)	67.4 (4.7)
Sex at birth, %		
Female	22	4
Male	78	96
Race and Ethnicity, %		
Black, non-Hispanic	47	22
Hispanic, any race	3	6
White, non-Hispanic	46	70
Other, non-Hispanic	4	2
Household income /year *, %		
≤$30,000	41	26
$30,001–$75,000	30	45
>$75,000	30	29
Education *, %		
Basic (Less than high school)	7	1
Intermediate (High school)	42	36
Advanced (At least some college)	51	63
Smoking status, %		
Never	27	30
Former	55	62
Current	18	8
Current alcohol use, %	69	77
Diabetes mellitus, %	25	26
Hypertension, %	57	58
**HIV-related**		
Current CD4 cells/µL *, %		
<200	1	
200–500	29	
>500	70	
Nadir CD4 cells/µL, %		
<200	36	
200–500	54	
>500	10	
Current HIV RNA copies/µL among detectable, weighted median (IQR)	40 (30, 52)	
<200	97	
Years of HIV infection, weighted mean (SD)	42 (5.3)	
**Health insurance and provider access**		
Has an eye care provider *, %	81	87
Health insurance, %		
Private Insurance	53	45
Medicaid	8	8
Medicare	35	38
Other coverage (VA, Other health insurance specified)	1	9
Unknown (reported they have insurance not the type of insurance)	3	0

Abbreviations. HIV: Human Immunodeficiency Virus; PLWH: people living with HIV; PWOH: people living without HIV. Note: all percentages are from the weighted data. * Missing data. Participants with missing data on education and income, and have eye care providers: 1; current CD4 cell count: 2.

**Table 2:** Visual function, ocular condition, and OCTA findings in PLWH and PWOH.

	PLWH N = 74	PWOH N = 65
**Visual Function**		
VA Impairment, %		
Distance Presenting VA Impairment	4	1
Distance Corrected VA Impairment *	0	3
Near Presenting VA Impairment	9	4
CS Impairment, %	3	4
Processing Speed * (ms) weighted mean (SD)	18.4(8.5)	18.4(8.5)
Divided Attention * (ms) weighted mean (SD)	138.7(62.9)	147.4(89.7)
Selective Attention * (ms) weighted mean (SD)	222.1(87.9)	211.4(91.2)
**Ocular Condition**		
Uncorrected refractive error, %	15	5
VA improvement by refraction *, %	63	64
IOP > 21 mmHg *, %	6	4
Has an eye care provider *, %	81	87
Dilation *, %		
One eye	44	52
Both eyes	47	46
Had cataract surgery, %	26	7
Cataract grading, %		
Nuclear grading (≥2)	14	12
Posterior Subcapsular * (≥3 mm)	0	1
Cortical grading* (≥4/16)	12	1
Cataract *: • Had cataract surgery or • Nuclear grading (≥2) or • Posterior Subcapsular (≥3 mm) or • Cortical grading (≥4/16)	49	20
Meibomian gland dysfunction *, %	20	8
Conjunctival redness *, %	11	4
Tear Insufficiency by Schirmer II test, %		
Mild	19	15
Moderate	21	16
Severe	16	19
Signs of Dry Eye Disease *: % • Meibomian gland dysfunction or • Mild or worse tear insufficiency	63	51
Pigments on endothelium *, %	3	7
Irregular pupil size or shape *, %	3	1
Active uveitis *, %	0	0
Retinitis *, %	4	0
Glaucomatous optic neuropathy *, %	19	25
Age-related macular degeneration *, %	1	4
Other retinal pathology *, %	4	1
Epiretinal membrane *, %	9	15
Pre-proliferative DR *, %	1	4
Proliferative DR *, %	0	0
Macular Edema *, %	1	0
**OCT retinal measurements**		
Optic nerve head nerve fiber layer thickness * (µm), weighted mean (SD)	90(11)	88(15)
Superficial capillary plexus vessel density * (%), weighted mean (SD)	45(4)	44(4)
FAZ * (mm^2^), weighted mean (SD)	0.3(0.1)	0.2 (0.09)

Abbreviations. PLWH: people living with HIV; PWOH: people living without HIV; VA: visual acuity; CS: contrast sensitivity; IOP: Intraocular pressure; DR: diabetic retinopathy; OCT: optical coherence tomography; FAZ: foveal avascular zone. Definitions. VA Impairment: visual acuity worse than 20/40 in the better eye; CS Impairment: contrast sensitivity worse than 1.50 log CS in the better eye; Other retinal pathology included macular degeneration other than age-related macular degeneration, epiretinal membrane, pre-proliferative DR, proliferative DR, and macular edema. * Missing data. Participants with missing data on distance corrected VI, cataract, and dilation: 1; uncorrected refractive error, cataract surgery, conjunctival redness, pigments on endothelium, and irregular pupil size or shape: 2; high IOP and Meibomian gland dysfunction: 4; posterior subcapsular and cortical grading: 10; cataract, signs of dry eye disease, and tear insufficiency: 8; active uveitis, retinitis, glaucomatous optic neuropathy, age-related macular degeneration, other macular degeneration, epiretinal membrane, pre-proliferative DR and proliferative DR, and macular edema: 5; optic nerve head nerve fiber layer thickness: 24; superficial capillary plexus vessel density: 27; FAZ: 29; processing speed: 6; divided and selective attention, UVOF: 7.

## Data Availability

Data associated with this paper are available by request from the MWCCS: https://statepi.jhsph.edu/mwccs/work-with-us/ (MWCCS data release date: 15 October 2024).
